# Marine exotic isopods from the Iberian Peninsula and nearby waters

**DOI:** 10.7717/peerj.4408

**Published:** 2018-02-27

**Authors:** Gemma Martínez-Laiz, Macarena Ros, José M. Guerra-García

**Affiliations:** 1 Laboratorio de Biología marina, Departamento de Zoología, Facultad de Biología, Universidad de Sevilla, Seville, Spain; 2 Departamento de Biología, CASEM. Facultad de Ciencias del Mar y Ambientales, Campus Universitario de Puerto Real, Puerto Real, Cadiz, Spain

**Keywords:** Isopoda, Exotic species, Recreational boating, Iberian Peninsula, Strait of Gibraltar

## Abstract

Effective management of marine bioinvasions starts with prevention, communication among the scientific community and comprehensive updated data on the distribution ranges of exotic species. Despite being a hotspot for introduction due to numerous shipping routes converging at the Strait of Gibraltar, knowledge of marine exotics in the Iberian Peninsula is scarce, especially of abundant but small-sized and taxonomically challenging taxa such as the Order Isopoda. To fill this gap, we conducted several sampling surveys in 44 marinas and provide the first comprehensive study of marine exotic isopods from the Iberian Peninsula, the southern side of the Strait of Gibraltar (northern Africa) and the Balearic Islands. Exotic species included *Ianiropsis serricaudis* (first record for the Iberian Peninsula and Lusitanian marine province), *Paracerceis sculpta* (first record for the Alboran Sea ecoregion), *Paradella dianae*, *Paranthura japonica* (earliest record for the Iberian Peninsula) and *Sphaeroma walkeri*. Photographs with morphological details for identification for non-taxonomic experts are provided, their worldwide distribution is updated and patterns of invasion are discussed. We report an expansion in the distribution range of all species, especially at the Strait of Gibraltar and nearby areas. *Ianiropsis serricaudis* and *Paranthura japonica* are polyvectic, with shellfish trade and recreational boating being most probable vectors for their introduction and secondary spread. The subsequent finding of the studied species in additional marinas over the years points at recreational boating as a vector and indicates a future spread. We call for attention to reduce lags in the detection and reporting of small-size exotics, which usually remain overlooked or underestimated until the invasion process is at an advanced stage.

## Introduction

In marine ecosystems, the spread of exotic species is one aspect of global change (Occhipinti-Ambrogi, 2007) and shipping is known to be the main vector for both primary introduction and secondary spread, via ballast water or biofouling (Ruiz et al., 2000). In the Mediterranean Sea, the most invaded sea in Europe, introduction events increased enough to more than double the total number of exotic species between 1970 and 2015, with intensification of commercial shipping being the main reason (Galil, Marchini & Occhipinti-Ambrogi, 2016; Galil et al., 2017). These introductions can have diverse and complex impacts, including significant biological harm and socioeconomic costs (Carlton, 2002; Molnar et al., 2008). Notorious examples are the cases of the European green crab *Carcinus maenas* (Malacostraca: Decapoda) and the Chinese mitten crab *Eriocheir sinensis* (Malacostraca: Decapoda), both being aggressive competitors for native species, affecting aquaculture facilities and harvests and causing structural damage to river banks (Klassen & Locke, 2007; Veilleux & De Lafontaine, 2007). Similarly, the Japanese amphipod *Caprella mutica* (Malacostraca: Amphipoda), despite having a much smaller size and being less notorious, also achieved a globally widespread distribution in a relatively short timeframe, as well as causing malfunctioning to pumps and fouling biomass to cages in aquaculture facilities (Boos, Ashton & Cook, 2011).

The Order Isopoda includes marine, brackish, freshwater and terrestrial species, occupying areas from the desert to the deep sea. It comprises 379 genera in 37 families of marine isopods inhabiting all marine habitats including temperate realms, tropical regions and polar seas (Espinosa-Pérez & Hendrickx, 2006; Poore & Bruce, 2012). They show a variety of feeding modes including detritus feeders, carnivores, parasites, filter feeders and browsers. They also have been attributed a certain economic impact, being either diet for fish or their ectoparasites and thus potentially affecting commercial stocks, as well as causing damage of wharf and timber structures (see Poore & Bruce, 2012). Indeed, they are also great invaders around the world (Galil, Clark & Carlton, 2011; Chapman & Carlton, 1991; Orensanz et al., 2002), and are potentially transportable by a number of vectors such as vessels, aquaculture, live seafood, contaminated gear and footwear, marsh restoration and floating plastic debris, among others (Carlton, 2011). For example, the invasive burrowing isopod *Sphaeroma quoyanum* has caused several impacts in California saltmarshes by reducing sediment stability and increasing erosion, ultimately converting this habitat to mudflats (Talley, Crooks & Levin, 2001). Nevertheless, this group poses limitations for a correct assessment of exotics, mainly because they are small and taxonomically challenging; it is easy to find cases of misidentifications, inaccurate data, cryptic species or erroneous assignment of introduced status (see Xavier et al., 2009; Carlton, 2011; Marchini, Ferrario & Occhipinti-Ambrogi, 2016a). They can thus remain undetected for many years even if they pose a threat to surrounding species (Carlton, 2011); and this kind of data-gaps and inaccuracies are some of the main factors hampering a correct management of bioinvasions (see Ojaveer et al., 2015; Galil, Marchini & Occhipinti-Ambrogi, 2016). Reports of updated distribution of exotics and arrivals in new areas are vital to overcome these obstacles. For example, in the Iberian Peninsula, Baleares and northern coast of Africa, studies dealing with Isopoda include the catalogs published by Castelló (1986), Castelló & Carballo (2001), Castellanos, Hernández-Vega & Junoy (2003) and Junoy & Castello (2003); however, no further revisions or checklists about exotic isopods are available at present. This is an urgent issue to solve, since the Iberian Peninsula is highly threatened by exotic species introduction due to its biogeographical position; it bears intense maritime traffic all around, with numerous shipping routes converging at the Strait of Gibraltar (see Seebens, Gastner & Blasius, 2013). Approximately 60,000 vessels transit the Strait each year; and it serves as gateway connecting areas like the Mediterranean Sea, West Africa, the Caribbean, northern Europe and Australia (Gibraltar Port Authority, 2017; Gibraltar Port marina staff, pers. comm., 2017), thus being a high-risk pathway for exotic species (see Drake & Lodge, 2004).

In marine bioinvasions, once a species has established in a new location, its effects are most often irreversible (Streftaris, Zenetos & Papathanassiou, 2005). Well-known examples are the algae *Caulerpa taxifolia* and the zebra mussel *Dreissena polymorpha*. This means that measures need to first focus on prevention and early detection rather than eradication (Simberloff, 2009; Roy et al., 2014). Monitoring surveys are an integral tool in here (see Bishop & Hutchings, 2011), and marinas are suitable spots for this purpose. While being underestimated in the past (Minchin et al., 2006; Clarke-Murray, Pakhomov & Therriault, 2011; Clarke-Murray, Therriault & Pakhomov, 2013), they have proved to be hotspots for introduction and subsequent spreading of non-indigenous species (thereafter NIS) (Cohen et al., 2005; Glasby et al., 2007; Floerl et al., 2009; Lacoursiére-Roussel et al., 2012; Ros et al., 2014; Foster et al., 2016; Ferrario et al., 2016a; Ferrario et al., 2017). As such, several sampling surveys along the marinas of the Iberian Peninsula, the Baleares Islands and the northern coast of Africa were carried out from 2011 to 2017, exploring a wide range of fouling substrates, in order to provide the first comprehensive study of marine exotic isopods in the Iberian Peninsula and adjacent waters, and discuss potential pathways and vectors of introduction.

## Material & Methods

Examined material was collected during several sampling surveys carried out from 2011 to 2017, in order to study the fouling epifauna in 44 marinas around the Iberian Peninsula, the Southern side of the Strait of Gibraltar (northern Africa) and Baleares. Marina choice was based on its vessel traffic and popularity as tourist locality (see Table 1 including number of berths and population density). Data for number of berths was obtained from the FEAPDT (Federación Española de Puertos Deportivos y Turísticos: http://www.feapdt.es) and from the IPTM (Instituto Portuário e dos Transportes Marítimos: http://www.atlanticstrategy.eu/en/partners/iptm-instituto-portu%C3%A1rio-e-dos-transportes-mar%C3%ADtimos-ip). Census data for the locality to which each marina belongs was obtained from the National Statistical Systems of Spain (http://www.ine.es), Portugal (http://www.ine.pt) and Morocco (http://www.hcp.ma) (Ros, Vázquez-Luis & Guerra-García, 2015). In 2011, the abundant bryozoans *Bugula neritina* and *Amathia verticillata*, together with its associated epifauna, were collected from marinas around the Peninsula and the Strait of Gibraltar (Ros, Vázquez-Luis & Guerra-García, 2015). Additionally, two monitoring programmes were carried out along the year 2012 in Puerto de Palma marina (Palma de Mallorca, Balearic Islands) and Puerto América marina (Cádiz), in which the substrates *Amathia verticillata* and *Eudendrium* sp. were sampled*.* Finally, a sampling survey was carried out during 2017 along the southern coast of the Iberian Peninsula to cover the main marinas of Andalusian coast. This area was selected as convergence zone between the Mediterranean Sea and the Atlantic Ocean, bearing a big gateway for marine introductions as it is the Strait of Gibraltar. Fouling organisms growing on artificial hard substrate including pontoons, ropes, wheels, buoys and ship hulls were sampled. These included red and green algae, hydroids, bryozoans, ascidians and mollusks plus their associated mobile epifauna. Samples were hand-collected, fixed in 90% ethanol and taken to the laboratory. Isopods were sorted, counted and identified to species level following updated literature on the group. Valid alien status was assigned following the European Environmental Agency criteria EEA, 2012, and valid human-mediated introduction was assessed based on Chapman & Carlton (1991). Photographs of full specimens and morphological parts of interest were taken using the camera Sony DSC-WX50. Worldwide distribution maps were developed using QGIS 1.8.0 Lisboa (QGIS, 2015), and shapefiles of marine ecoregions were obtained from http://maps.tnc.org/gis_data.html (accessed 20/08/2017). Voucher material of each species was deposited in the Museo Nacional de Ciencias Naturales (MNCN,CSIC), Madrid, Spain. The rest of the material was kept in the Laboratorio de Biología Marina, University of Seville, Spain.

## Results

Five exotic marine isopods were found on fouling communities associated to marinas: *Ianiropsis serricaudis*, *Paracerceis sculpta*, *Paradella dianae*, *Paranthura japonica* and *Sphaeroma walkeri* (Table 1). From the sampled marinas, 53% hosted exotic isopods, with marinas around the Strait of Gibraltar being the most invaded ones (e.g., Cádiz Bay hosting four of the five species) and *Paracerceis sculpta* the most widespread species. Out of the 14 marinas that were sampled in 2011/2012 and again in 2017, seven (50%) had increased the number of exotic species, sometimes by 200% or more (see Table 1). We provide the first record of *Ianiropsis serricaudis* for the Iberian Peninsula and the Lusitanian marine province, the first record of *Paracerceis sculpta* for the Alboran Sea ecoregion, and the earliest (2011) record of *Paranthura japonica* from the Iberian Peninsula. We report an extension in the distribution range for all species along the coasts of the Iberian Peninsula and adjacent waters.

**Table 1:** Data of sampled marinas and presence of exotic isopods. List of sampling localities (stations), coordinates, number of marina berths, population density (mean number of people per km^2^) and sampling year of each sampled marina. Exotic isopod species present in each marina are indicated; Is, *Ianiropsis serricaudis*; Ps, *Paracerceis sculpta*; Pj, *Paranthura japonica*; Pd, *Paradella dianae* and Sw, *Sphaeroma walkeri*; “–”, no exotic isopods or no isopods at all present; “blank”, no sampled. In grey, the cases in which an increased in exotic isopod species was found in 2017.

Station (St)	Coordinates	No. of marina berths	Population density	Exotic isopods 2011/2012	Exotic isopods 2017
1. Santander	43.45°N, 3.82°W	900	5,176	–	
2. Gijón	43.54°N, 5.67°W	779	1,527	–	
3. Ferrol	43.48°N, 8.26°W	250	883	Is	
4. A Coruña	43.37°N, 8.40°W	700	6,503	–	
5. Nazaré	39.59°N, 9.07°W	52	180	–	
6. Cascais	38.69°N, 9.42°W	650	1,832	–	
7. Sines	37.95°N, 8.87°W	230	67	–	
8. Albufeira	37.08°N, 8.27°W	475	251	–	
9. Faro	37.01°N, 7.94°W	300	289	Ps	Ps
10. Isla Cristina	37.19°N, 7.34°W	231	448	–	Ps
11. El Rompido	37.22°N, 7.13°W	387	85	–	–
12. Chipiona	36.74°N, 6.43°W	447	573	–	Ps, Pj
13. Rota	36.62°N, 6.35°W	209	347	Ps	Ps, Pj, Pd
14.1 Cádiz, Puerto América	36.54°N, 6.38°W	319	10,154	Ps	Ps, Pd, Sw, Pj
14.2 Cádiz, V. de Levante	36.52° N, 6.30° W	270	10,154		Ps, Pj
15. Sancti Petri	36.40°N, 6.21°W	94	389	–	–
16. Conil	36.29°N, 6.14°W	97	245	Ps	
17. Barbate	36.19°N, 5.93°W	314	160	–	Ps, Pd
18. La Línea	36.16°N, 5.36°W	624	3,370	Ps	–
19. Fuengirola	36.54°N, 4.62°W	275	7,145	–	Ps
20. Benalmádena	36.60°N, 4.51°W	1,140	2,373	–	
21. Málaga	36.72°N, 4.41°W	107	1,437	–	–
22. Caleta Vélez	36.75°N, 4.07°W	277	488	Pd	Pd
23. Motril	36.72°N, 3.53°W	193	555	–	Pd
24. Almerimar	36.70°N, 2.79°W	1,100	371	–	
25. Roquetas	36.76°N, 2.61°W	237	1,506	–	
26. Almería	36.83°N, 2.46°W	277	643	–	–
27. Carbonera	36.99°N, 1.90°W	48	86	–	
28. Torrevieja	37.97°N, 0.68°W	570	1,430	Ps	
29. Alicante	38.34°N, 0.49°W	400	1,661	Ps	
30. Dénia	38.85°N, 0.11°W	300	676	Ps	
31. Valencia	39.43°N, 0.33°W	206	5,928	Ps	
32. Borriana	39.86°N, 0.07°W	713	126	–	
33. Oropesa Mar	40.08°N, 0.13°W	668	126	–	
34. Benicarló	40.42°N, 0.43°W	293	126	Ps, Pj	
35. Tarragona	41.11°N, 1.25°W	441	2,436	–	
36. Vilanova Geltrú	41.21°N, 1.73°W	812	1,976	–	
37. Barcelona	41.38°N, 2.18°W	200	16,449	Pj	
38. L’Estartit	42.05°N, 3.21°W	738	172	–	
39. Tánger	35.79°N, 5.81°W	500	229	–	
40. Ceuta	35.89°N, 5.32°W	325	4,229	Ps	
41. MSmir	35.75°N, 5.34°W	450	283	Ps	
42. M’Diq	35.68°N, 5.31°W	120	283	Ps	
43. Puerto de Palma	39.34°N, 2.38°E	996	1,931	Pj	

**Table:** 

**Figure 1:**
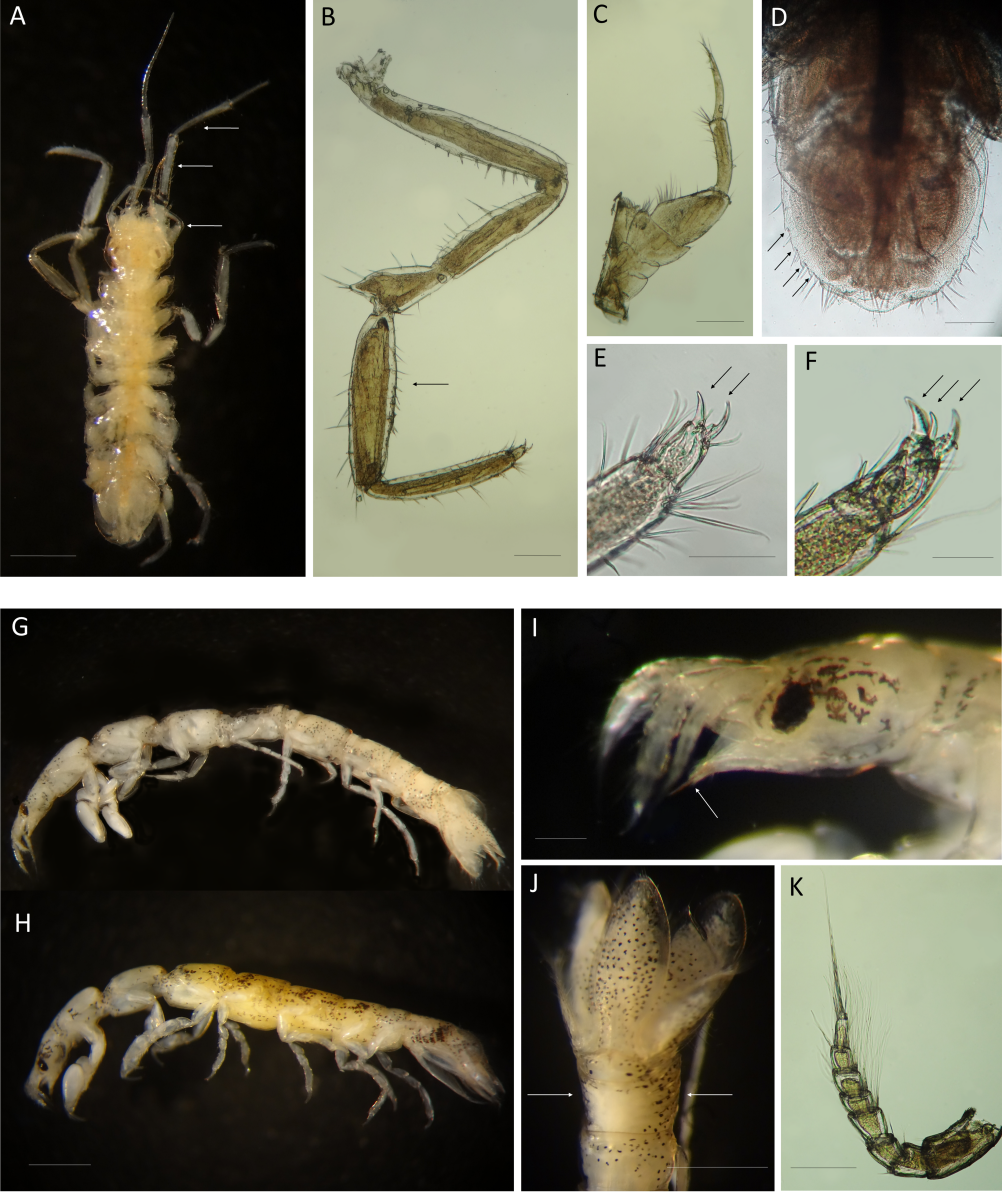
Useful morphological details for identification of marine exotic isopods on fouling communities associated to marinas (Families Janiridae and Paranthuridae). Families Janiridae** (A–F) and Paranthuridae (G–K). *Ianiropsis serricaudis* from La Graña marina (Ferrol, Spain) (St 3); male dorsal view (A), Pereopod 1(B), maxilliped (C), pleotelsonic dentation (D), two claws on pereopod 1(E), three claws on pereopod 7(F). *Paranthura japonica* from Puerto America marina (Cádiz, Spain) (St 14.1); male lateral view (G), female (H), female pointed mouthparts (I), semi-segmented pleon (J), antenna 1 (K). Bar 1 mm: A,G,H,J. Bar 0.2 mm: B,C,D,I,K. Bar 0.05 mm: E,F. Arrows show specific morphological details described in the text.

**Suborder Asellota Latreille, 1802**
**Family Janiridae G.O. Sars, 1897**
Genus *Ianiropsis* G.O. Sars, 1897a
*Ianiropsis serricaudis* (Gurjanova, 1936)
(Figs. 1A–1F)

*Janiropsis serricaudis* Gurjanova, 1936, pg. 251–252, Fig. 1

*Ianiropsis notoensis* Nunomura, 1985, pg. 130–132, Figs. 7–8

**Figure 2:**
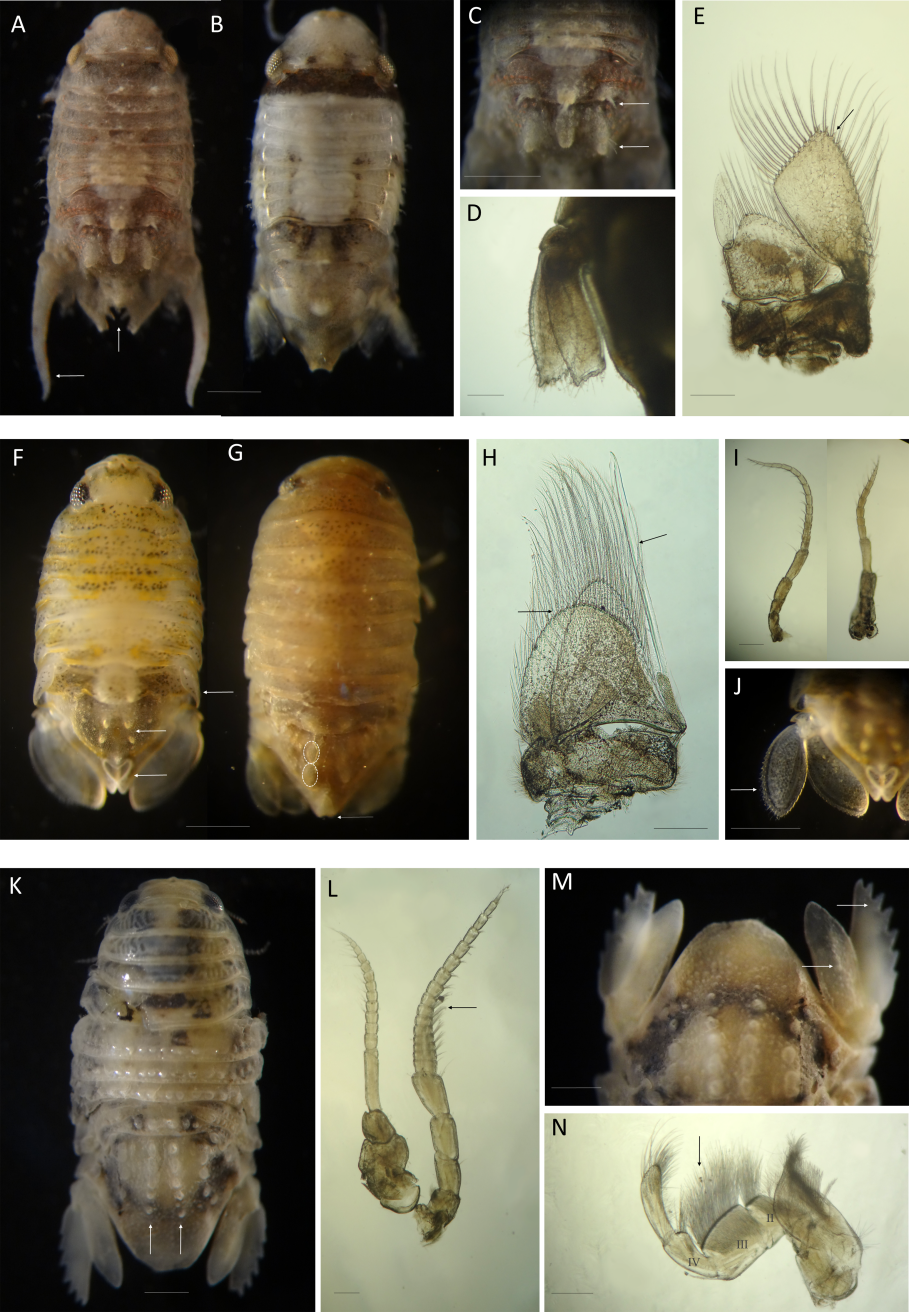
Useful morphological details for identification of marine exotic isopods on fouling communities associated to marinas (Family Sphaeromatidae). Family Sphaeromatidae. *Paracerceis sculpta* from Barbate marina (Cádiz, Spain) (St 17)** (A–E); male dorsal view (A), female (B), male pleotelson (C), female uropods (D), male pleopod 2 (E). *Paradella dianae* male from Barbate marina (Cádiz, Spain) (St 17) and female from Caleta-Vélez marina (Málaga, Spain) (St 22) (F–J); male dorsal view (F), female (G), male pleopod 2 (H), male antenna (left) and antennule (right) (I), male uropods (J). *Sphaeroma walkeri* from Puerto America marina (Cádiz, Spain) (St 14.1) (K–N); female dorsal view (K), female antennule (left) and antenna (right) (L), female pleotelson and uropods (M), female maxilliped (N). Bar 1 mm: A, B, E, F, G, K, M. Bar 0.2 mm: C, D, I, J, L, N. Arrows and dashed circles show specific morphological details described in the text.

**Figure 3:**
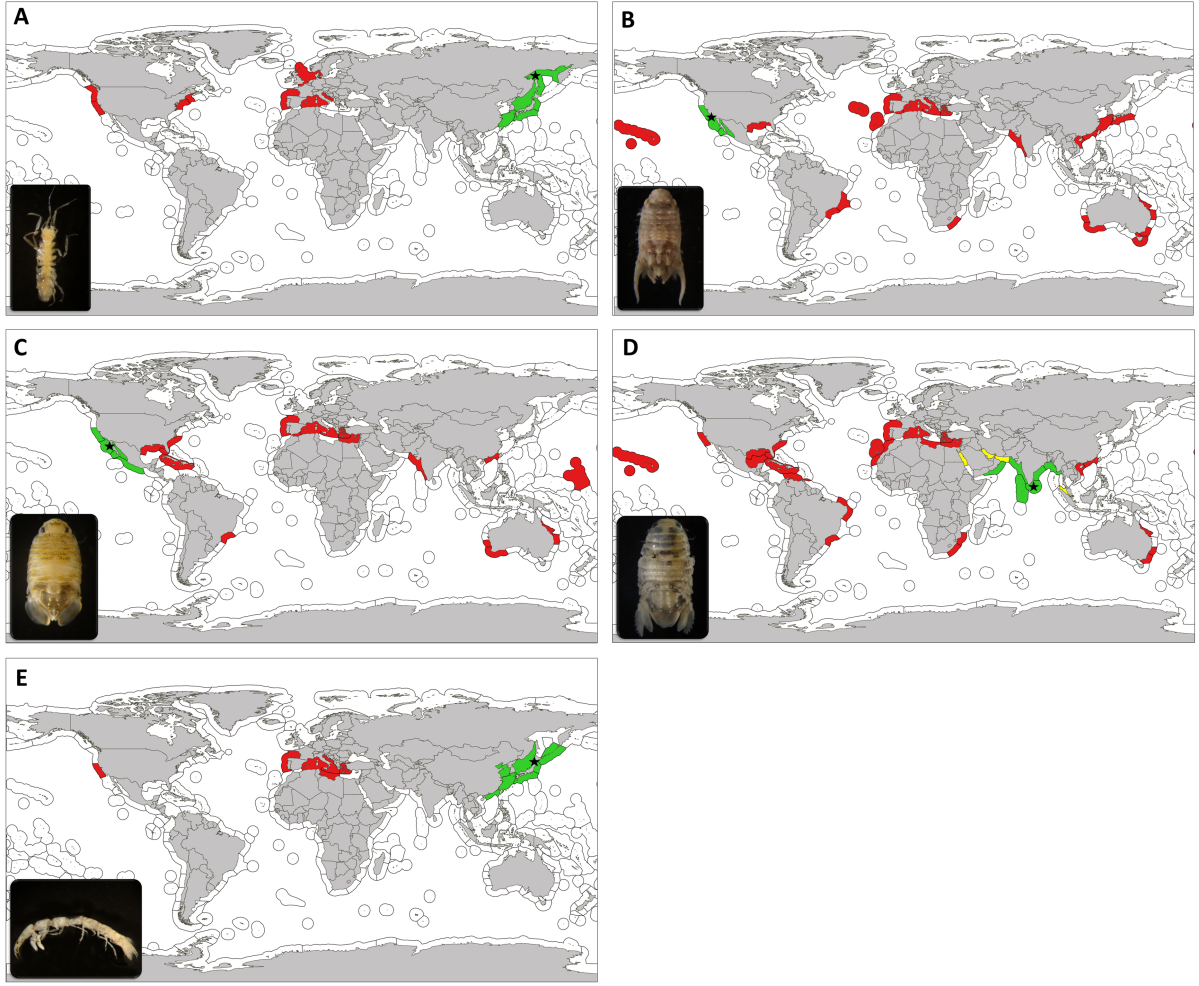
Updated worlwide distribution of marine exotic isopods found in marinas of the Iberian Peninsula and nearby waters. Updated worldwide distribution of *Ianiropsis serricaudis* (A), *Paracerceis sculpta* (B), *Paradella dianae* (C), *Sphaeroma walkeri* (D) and *Paranthura japonica* (E), divided by marine ecoregions. Areas in green show the native range, areas in red show introduction range and those in yellow indicate localities where we consider the species to be cryptogenic. Type locality is indicated with a star. Marine ecoregions following Spalding et al. (2007).

*Ianiropsis serricaudis* Kussakin, 1962, pg. 49–50, Fig. 25; Kwon & Heon, 1990, pg. 195, Fig. 2B; Shimomura & Kajihara, 2001, pg. 48; Yokoyama & Ishihi, 2007, pg. 132; Doti & Wilson, 2010, pg. 16; Heiman & Micheli, 2010, Table 1; McIntyre et al., 2013, pg. 30; Wells et al., 2014, pg. 6 and 19; Hobbs et al., 2015, pg. 179–182, Figs. 1– 5; Marchini, Ferrario & Occhipinti-Ambrogi, 2016a; Marchini, Ferrario & Occhipinti-Ambrogi, 2016b, pg. 333, Figs. 2–3; Ferrario et al., 2017, pg. 4–6; Ulman et al., 2017, pg. 9, Table 2, pg. 13, Table 5, pg. 26.

*Ianiropsis* sp. Pederson et al., 2005, pg. 12.

*Ianiropsis* sp. Faasse, 2007, pg. 126, Fig. 2.

Material examined (total: 139 specimens): **St3**: 2 males (MNCN 20.04/11439), 18 males and 119 females clinging on bryozoan *Bugula neritina*, floating pontoons, 07/05/2011.

Taxonomical remarks: The genus *Ianiropsis* is similar to *Janira* and *Carpias:* three claws on walking legs, coxae visible in dorsal view and usually can only be definitely identified from the males. *Ianiropsis* can be distinguished from the other two by bearing an elongated carpus of male pereopod I (Fig. 1B), instead of enlarged or swollen propodus and carpus (*Carpias*) or not elongated propodus and carpus at all (*Janira*) (Wilson & Wägele, 1994). Our specimens showed the features pointed out by Doti & Wilson (2010), Hobbs et al. (2015), Marchini, Ferrario & Occhipinti-Ambrogi (2016a) and Marchini, Ferrario & Occhipinti-Ambrogi (2016b) for *I. serricaudis*: (i) antennal peduncle segments 6 and 7 particularly elongated relative to the overall length of the antennae (Fig. 1A); (ii) head anterior margin in dorsal view concave; (iii) distinctive maxilliped palp of adult male, projecting substantially, enough to be visible on head in dorsal view (Figs. 1A, 1C) (Doti & Wilson, 2010); (iv) dactylus of pereopod 1 bearing two claws while that of pereopod 7 bearing three (Figs. 1E, 1F respectively); (v) four marginal denticles on pleotelson (Fig. 1D).

Ecological remarks: The species presents a cosmopolitan distribution according to Doti & Wilson (2010), inhabiting mostly temperate to cold temperate coastal waters. In its native range it is distributed under rocks, on sponges, ascidians, coralline and brown algae, and rhizoids of kelp *Laminaria*, in water temperatures from 1.8 °C to 24 °C (Gurjanova, 1936; Kussakin, 1962, Kussakin, 1988).

**Table:** 

**Suborder Cymothoida Wägele, 1989**
**Family Paranthuridae Menzies & Glynn, 1968**
Genus *Paranthura* Spence Bate & Westwood, 1866
*Paranthura japonica* Richardson, 1909
(Figs. 1G–1K)

*Paranthura japonica* Richardson, 1909, pg. 77–78, Figs. 4–5; Kussakin, 1975, pg. 53, 67; Nunomura, 1975, pg. 28–31, Figs. 10–12; Nunomura, 1977, pg. 86–87, Fig. 12; Che & Morton, 1991, pg. 205, Table 4; Moshchenko & Zvyagintsev, 2004, pg. 8, 13, table 2, Fig. 2; Li, 2003, pg. 139, table 1, pg. 156, table 3; Cohen et al., 2005, pg. 1001, Appendix A table; Yamada et al., 2007, pg. 346–348, 352, table 2; Zhang et al., 2009, pg. 306, table 2, 308; Wang, Ren & Xu, 2010, pg. 610, 612, table 3; Frutos, Sorbe & Junoy, 2011, pg. 17; Lavesque et al., 2013, pg. 215–218, Fig. 2; Marchini et al., 2014, pg. 545–551, Figs. 2–5; Marchini, Ferrario & Minchin, 2015, pg. 358, Fig. 4; Lorenti et al., 2016, pg. 12792–12794, Figs. 2–4; Tempesti et al., 2016, Fig. 1; (Ferrario et al., 2016b), pg. 224, 225, table 1; Dailianis et al., 2016, pg. 609, table 1, pg. 615, Fig. 9; Ferrario et al., 2017, pg. 4–5,7; Ulman et al., 2017, pg. 9, Table 2, pg. 13, Table 5, pg. 26, 27, 36.

*Paranthura* sp. (Cohen & Carlton, 1995), pg. 84, 146, Table 1, pg. A4-2, Table 1.

Material examined (total: 139): **St12:** Two females and two juveniles from fouling community on floating structures (pontoons, ropes and buoys), 01/07/2017. **St13:** Six males, 13 females and 24 juveniles from Corallinaceae algae and green algae, 13/05/2017. **St14.1**: Four females and one juvenile on *B. neritina*, one male and one juvenile on *Eudendrium* sp., and one male and two female on Coralline algae, floating pontoons, 14/05/2017; four males, 12 females and 33 juveniles from fouling community on floating pontoons, 14/05/2017; one male and two females (MNCN 20.04/11443), three males six females and 16 juveniles collected from fouling community on floating structures, 02/07/2017. **St14.2** One female and one juvenile from fouling substrates, floating structures, 02/07/2017. **St34**: One juvenile on *A. verticillata*, floating pontoons, 27/06/2011. **St37:** One female and one juvenile on *A. verticillata*, floating pontoons, 26/06/2011. **St43**: One female on *Eudendrium* sp., floating pontoons, 09/2012.

Taxonomical remarks: the specimens match the descriptions by Richardson (1909), redescriptions by Nunomura (1975), Lavesque et al. (2013) and Lorenti et al. (2016). They display stinging mouthparts, typical of the family Paranthuridae (Fig. 1I), and a particular combination of characters that distinguish it from other known Japanese *Paranthura* species. These are: eyes well developed composed of less than 17 dark ommatidia; anterolateral angles of cephalon exceeding rostral projection; antenna 1 with 8 distinct articles (Fig. 1K); pereonite 6 shorter than pereonite 5; short pleotelson barely exceeding the tip of uropods; and particularly, semi-segmented pleon, with pleonites fused in the middle of their dorsal region but distinct at their sides, which allow to clearly identify *P. japonica* (Fig. 1J) (Lavesque et al., 2013; Lorenti et al., 2016).

Ecological remarks: *Paranthura japonica* is reported from coastal transitional ecosystems, such as lagoons, estuaries, and mangroves (Lorenti et al., 2016). It adapts to a wide range of habitats including sandy bottoms in seagrass beds (*Zostera*), among algae (*Sargassum*) and in mussel beds and oyster reefs (Golovan & Malyutina, 2010; Lavesque et al., 2013). It is a successful colonizer of boat wreck and pontoons fouling, inhabiting crevices and free spaces between colonial animals as well as burrows made by other organisms (Cadien & Brusca, 1993; Kussakin 1982; Lorenti et al., 2016).

**Table:** 

**Suborder Sphaeromatidea Wägele, 1989**
**Family Sphaeromatidae Latreille, 1825**
Genus *Paracerceis* Hansen, 1905
*Paracerceis sculpta* (Holmes, 1904)
(Figs. 2A–2E)

*Dynamene sculpta* Holmes, 1904, pg. 300–302, pl. XXXIV, Figs. 1–7.

*Cilicaea sculpta* Richardson, 1905, pg. 318–319, Fig. 349.

*Paracerceis sculpta* Menzies, 1962, pg. 340–341, Fig. 2; Miller, 1968, pg. 14, Fig. 3; Schultz, 1969, pg 120, Fig. 167; Rezig, 1978, pg. 175; Brusca, 1980, pg, 226, Fig. 12.5–12.6; Pires, 1981, pg. 219–220; Harrison & Holdich, 1982, pg. 440–441, Fig. 10; Pires, 1982, pg. 45,53, Fig. 26–27; Forniz & Sconfietti, 1983, pg. 197–203, Figs. 1–2; Forniz & Maggiore, 1985, pg. 780; Shuster, 1987, pg. 321–323, Figs. 1, 3; 1990, pg. 390, Fig. 1; 1992, pg. 232–234, Fig. 1; Rodríguez, Drake & Arias, 1992, pg. 95–96, Figs. 2A, 2B; Loyola e Silva, Masunari & Dubiaski-Silva, 1999, pg. 109–123, Figs. 1–18; Yasmeen & Javed, 2001, pg. 43–48, Figs. 1–3; Yu & Li, 2001, pg. 48–49; Hewitt & Campbell, 2001, pg. 925–934; Espinosa-Pérez & Hendrickx, 2002, pg. 1172–1176, Fig. 2C; Ariyama & Otani, 2004, pg. 54–55, Figs. 2A–2E; Yasmeen & Yousuf, 2006, pg. 116–118, Fig. 3; Brusca, Coelho & Taiti, 2007, pg. 518–19, 537–538, pl. 243A; Dailianis et al., 2016, pg. 609, Table 1, pg. 614, 615, Fig. 9; Marchini et al., 2017, pg. 3, Fig. 2; Ferrario et al., 2017, pg 5; Ulman et al., 2017, pg. 9, Table 2, pg. 11, Table 3, pg. 13, Table 5, pg. 28, 37; Ramalhosa et al., 2017, pgs. 1747–1749, pg. 1751–1752, Fig. 2, pg. 1755–1759.

*Sergiella angra* Pires, 1980a, pg. 212–218, Figs. 1–24; Pires, 1981, pg. 219–220.

*Paracerceis japonica* Nunomura, 1988, pg. 4–7, Figs. 3–4.

Material examined (total: 1,188): **St9**: Three females and five juveniles on *Bugula neritina*, three females on *Amathia verticillata*, floating pontoons, 11/05/2011; two males and two females (MNCN 20.04/11440), 14 males, 224 females and 192 juveniles on fouling substrates, floating structures (pontoons, ropes and buoys), 26/06/2017. **St10:** one female and four juveniles on fouling substrates, floating structures, 26/06/2017. **St12:** one female on fouling substrates, floating structures, 01/07/2017. **St13**: Three juveniles on *B. neritina*, one female and 10 juveniles on *A. verticillata,* floating pontoons, 17/05/2011; six juveniles on Coralline algae and green algae, floating pontoons, 13/05/2017. **St14.1**: One male, nine females, 19 juveniles on *B. neritina*, one male, 29 females, 23 juveniles on *A. verticillata*, floating pontoons, 17/05/2011; one female and six juveniles on *A. verticillata*, 12/2011; one juvenile on *A. verticillata*, one male and one female on hydrozoan *Eudendrium* sp., 05/2012; one juvenile on *A. verticillata*, 06/2012; one juvenile on *A. verticillata*, 07/2012; one female and 23 juveniles on *A. verticillata*, 08/2012; 15 females and 39 juveniles on *A. verticillata,* 09/2012; one female and five juveniles on *A. verticillata*, 10/2012; two females and nine juveniles on *A. verticillata* 11/2012; 8 females and 155 juveniles on fouling community, floating pontoons, 14/05/2017. **St14.2:** One male, six females and six juveniles on fouling substrates, floating structures, 01/07/2017. **St16**: One juvenile on *B. neritina*, floating pontoons, 17/05/2011. 18 females and 139 juveniles on fouling substrates, floating pontoons, /06/2017. **St17:** One male, 18 females and nine juveniles on fouling substrates, floating structures, 01∕07∕2017. **St18**: One juvenile on *B. neritina*, floating pontoons, 15/05/2011. **St19:** Two males, 18 females and 26 juveniles on fouling substrates, floating structures, 29/06/2017. **St28**: three females and seven juveniles on *B. neritina*, floating pontoons, 29/06/2011. **St29**: 8 females and 10 juveniles on *B. neritina*, floating pontoons, 29/06/2011. **St30**: Two juveniles on *A. verticillata*, floating pontoons, 28/06/2011. **St31**: One female and three juveniles on *B. neritina*, three females and seven juveniles on *A. verticillata*, floating pontoons, 28/06/2011. **St34:** five juveniles on *B. neritina*, six females and 54 juveniles on *A. verticillata*, floating pontoons, 27/06/2011. **St40**: Two juveniles on *B. neritina*, floating pontoons, 29/95/2011. **St41**: Seven juveniles on *B. neritina*, floating pontoons, 30/05/2011. **St42**: Two females and four juveniles on *B. neritina*, one juvenile on *A. verticillata*, floating pontoons, 30/05/2011.

Taxonomical remarks: Our specimens match the description and illustrations given by Menzies (1962), Rodríguez, Drake & Arias (1992), Brusca, Coelho & Taiti (2007) and Marchini et al. (2017). The genus *Paracerceis,* together with other *Cerceis*-like genera, can be distinguished by bearing pronounced marginal teeth on exopods of pleopods 1–3, especially obvious on pleopod 2 (Fig. 2E), in contrast to the crenulate margin or toothless margin on genera like *Dynamene*, *Sphaeroma* or *Paradella* (Fig. 2H) (Harrison & Ellis, 1991). Male specimens of *P. sculpta* collected in our survey presented a granulated pleon, with three tubercles on the anterior and posterior margins (Fig. 2C). The most peculiar feature are the greatly elongated cylindrical uropod exopods, which largely exceed margin of pleotelson, and a cleft posterior margin of pleotelson with three pairs of notches, indicative of *P. sculpta*. (Fig. 2A). Some variation was reported though regarding some minute characters of the pleotelson, for example the variation in setation of pleotelsonic and pleon tubercles (see Marchini et al., 2017). Our specimens bear dorsal tufts of setae on the pereonite, pleon and pleotelson tubercles (Fig. 2C), like populations from California (Brusca, Coelho & Taiti, 2007), Azores (Marchini et al., 2017) and Mediterranean Sea; and unlike other specimens with rather poor or absent setation from the Iberian Atlantic coast (Rodríguez, Drake & Arias, 1992), Brazil (Loyola e Silva, Masunari & Dubiaski-Silva, 1999) and Japan (Ariyama & Otani, 2004). Futhermore, the apex of male endopods are markedly pointed (Fig. 2A), similarly to the aforementioned specimens from Azores and Brazil. According to Shuster (1987), *P. sculpta* exhibits three distinct sexually mature male morphs in its native range, corresponding to different strategies for reproduction. The “*α*-males” are the largest, they bear distinct morphological characteristics of *Paracerceis* and defend a harem. The “*β*-males” are smaller; they resemble females and mimic their courtship behavior. The “*γ*-males” are the smallest; they resemble juveniles and attempt to sneak into *α*-male harems. Our populations were also examined in search of all morphs but only alpha males (6.55 ± 0.72 mm in length according to Shuster, 1992) were present.

Ecological remarks: The species inhabits coasts and lagoons of subtropical to temperate regions. It has been found in association with a range of substrates such as shallow water calcareous sponges (Richardson, 1905; Holmes, 1904; Brusca, 1980), *Sargassum* C. Agardh, 1820 and *Galaxaura* Lamouroux, 1816 in Brazil (Pires, 1981), barnacles (Loyola e Silva, Masunari & Dubiaski-Silva, 1999), oyster reefs (Munguia & Shuster, 2013) and bryozoans (Marchini, Ferrario & Minchin, 2015; Marchini et al., 2017). As a stenohaline species (thus low tolerance to freshwater conditions) it would have crossed the Panama channel via ballast water of ships (Espinosa-Pérez & Hendrickx, 2002).

**Table:** 

**Family Sphaeromatidae Latreille, 1825**
Genus *Paradella* Harrison & Holdich, 1982
*Paradella dianae* (Menzies, 1962)
(Figs. 2F–2J)

*Dynamenopsis dianae* Menzies, 1962, pg 342, Fig. 3; Glynn, 1968, pg 573; Schultz, 1969, pg 123

*Dynamenella dianae* Menzies & Glynn, 1968, pg 63, 113, Fig. 3; Glynn, 1970, pg 24, Figs. 9–10; Iverson, 1974, pg 166; Pires, 1980b, pg 134, Figs. 1–7

*Paradella dianae* Harrison & Holdich, 1982, pg 104, Fig. 6; Pires, 1982, pg. 45, 51–53, Figs. 21–23; Fox & Ruppert, 1985, pg. 317; Javed & Ahmed, 1987, pg. 216, Fig. 1; Kensley & Schotte, 1989, pg. 224–225, Figs. 98A–98C, pg. 266, 268, Table 6; Atta, 1991, pg. 213–217, Figs. 2,3; Rodríguez, Drake & Arias, 1992, pg. 96, Fig. 2; Nelson & Demetriades, 1992, pg. 648–649, Figs. 1–2, pg. 650, 652; Kensley, Nelson & Schotte, 1995, pg. 137, table 1, pg. 138, table 2; Kensley & Schotte, 1999, pg. 702–705, Figs. 4–5; Hass & Knott, 2000; pg. 461, table1; Castelló & Carballo, 2001, pg. 230; García-Guerrero & Hendrickx, 2004, pg. 1159; Wetzer & Bruce, 2007, pg. 39, 40, 42, 46 and 48; Çinar et al., 2008, pg. 1, 6–7, Table 2, pg. 12, 14; Knott and De Victor 2010, pg. 2–6, Figs. 1–3; Kirkim et al., 2010, pg. 102; Galil, 2011, pg. 231, Appendix 1, 236, Appendix 2, 242, Appendix 3, 384, table 1, 463, table 2; Ates et al., 2013, pg. 23; Doğan, Bakir & Katağan, 2015, pg. 857, 860–864, table 2; Kirkim, Özcan & Katagan, 2015, pg. 323–325, Fig. 2; Ferrario et al., 2017, pg. 4–5; Ulman et al., 2017, pg. 11, Table 3, pg. 28, 37.

*Paradella quadripunctata* Van Dolah, Knott & Calder, 1984, pg.52

Material examined (total: 49): **St13:** One male and two females (MNCN 20.04/11441), five females and 36 juveniles collected from Corallinaceae algae and green algae, floating pontoons, 13/05/2017. **St14.1:** Two juveniles collected from fouling community, floating pontoons, 14/05/2017. **St17:** One male collected from fouling community of floating structures (pontoons, buoys, ropes) 01/07/2017. **St23:** One female collected from fouling substrates, floating structures, 28/06/2017. **St22**: One female from fouling substrates, floating structures, 28∕06∕2017 and one female on *Bugula neritina*, floating pontoons, 03∕07∕11.

Taxonomical remarks: The specimens coincide with the characters explained by Menzies & Glynn (1968), Pires (1980b) (on *Dynamenella dianae*), Harrison & Holdich (1982), Wetzer & Bruce (2007) and Rodríguez, Drake & Arias (1992). The genus *Paradella* can best be identified by males having a distinct dorsally-directed, Y-shaped and posteriorly closed pleotelson foramen; long, tapering and basally fused penial processes, and a long and basally narrow appendix masculina that usually extends beyond the distal margin of the endopod (Fig. 2H) (Wetzel & Bruce, 2007). *Paradella dianae* males can be distinguished by the aforementioned Y-shaped or heart-shaped and posteriorly closed pleotelson foramen; by paired sub-median nodules on the pleon and two pairs of longitudinal carinae centrally arranged on the dorsal surface of the granulose pleotelson; and by large or expanded pereonite 7 coxae (Fig. 2F). *Paradella dianae* has ovate uropods, subequal in length, and with exopod and endopod of mature male large, with heavy, decidedly crenulate margins, with an evenly convex lateral margin on the uropodal exopod, characters that allow for its distinction from the similar congener *P. garsonorum* (Fig. 2J) (from Wetzer & Bruce, 2007; Harrison & Holdich, 1982). Uropoda of female are smaller than in male and apex of pleotelson has a slight reduced depression (Fig. 2G). Antennula flagellum has 11 artciles and antenna flagellum with 16 (Fig. 1I), similarly to the Arabian Sea and Cádiz specimens (Javed & Ahmed, 1987; Rodríguez, Drake & Arias, 1992) and unlike the Australian ones, which bear 12 and 13 articles respectively (Harrison & Holdich, 1982). Female submedian pair of tubercles are not completely fused (dashed circles in Fig. 1G), as indicated by Atta (1991) for Mediterrranean specimens. Size was consistent with populations previously reported from Cádiz Bay (Spain) (Rodríguez, Drake & Arias, 1992).

Ecological remarks: This isopod is commonly found amongst barnacles tests, intertidal green algae, bryozoans, empty polychaete tubes and rock oysters on rocks and man-made structures from upper to lower shore, in exposed and sheltered shores (Harrison & Holdich, 1982). It is known to survive at temperatures as low as 14 °C (Nelson & Demetriades, 1992), tolerant to some salinity variations, 31–38 pt. (García-Guerrero & Hendrickx, 2004) and also known to withstand heavy pollution (Pires, 1980b). It is protogynous hermaphrodite (Kensley & Schotte, 1999) and females can bear a peak of egg production during June (García-Guerrero & Hendrickx, 2004) or more than one peak in the introduced population (Nelson & Demetriades, 1992).

**Table:** 

**Family Sphaeromatidae Latreille, 1825**
Genus *Sphaeroma* Bosc, 1801
*Sphaeroma walkeri* Stebbing, 1905
(Figs. 2K–2N)

*Sphaeroma walkeri* Stebbing, 1905, pg. 31–33, pl. VII; 1910, pg. 220; 1917, pg. 444; Barnard, 1920, pg. 360; 1936, pg. 178; 1940, pg. 405; Omer-Cooper, 1927, pg. 240; Baker, 1928, pg. 49; Nierstrasz, 1931, pg. 192; Monod, 1931, pg. 36; Monod, 1933, pg. 198; Larwood, 1940, pg. 28; Pillai, 1955, pg. 132, pl. VI; Loyola e Silva, 1960, pg. 41, Figs. 6–7; Joshi & Bal, 1959, pg. 61–62; Menzies & Glynn, 1968, pg. 56, Fig. 23; Miller, 1968, pg. 8–11, Fig. 3; Glynn, 1972, pg. 286, Fig. 5; Carlton & Iverson, 1981: 31–46; Estevez & Simon, 1976, pg. 288; Harrison and Holdich 1984, pg. 279–282, Fig. 1; Jacobs, 1987, pg. 22–24, Fig. 6; Mak, Huang & Morton, 1985, pg. 75; Morton, 1987, pg. 504, Fig. 1; Kensley & Schotte, 1989, pg. 235, Fig. 101; Kussakin & Malyutina, 1993, pg. 117; Bruce, 1993, pg. 156, Fig. 1; Loyola e Silva, 1998, pg. 629; Ghani & Qadeer, 2001, pg. 871–872; Ramadan, Kheirallah & Abdel-salam, 2006, pg. 22, table 1; Galil, 2008, pg. 443, Fig. 1; Ben Amor, Ben Slaem & Ben Souissi, 2010, pg. 615, Fig. 1; Khalaji-Pirbalouty & Wägele, 2010, pg. 10–16, Figs. 6–10, 11D; Ben Amor, Rifi & Ben Soussi, 2015, pg. 37, Fig. 2; Ulman et al., 2017, pg. 9, Table 2, pg. 11, Table 3, pg. 13, Table 5, pg. 29.

Material examined (total: two females): **St14.1**: One female from fouling community, floating pontoons, 14/05/2017; one female (MNCN 20.04/11442) collected from fouling community, floating structures (pontoons, ropes, buoys), 02/07/2017.

Taxonomical remarks: The specimens coincide with the descriptions by Jacobs (1987), Khalaji-Pirbalouty & Wägele (2010) and Ben Amor, Rifi & Ben Soussi (2015). *Sphaeroma* can be distinguished from related genera like *Exosphaeroma* and *Lekanesphaera* by bearing a robust maxilliped, particularly the palp, articles II–IV without lobes and a fringe of robust, plumose setae on internal border of endite (Fig. 2N). The uropodal rami of *Sphaeroma* are subequal, usually reaching beyond the posterior margin of pleotelson and the external margin of exopod is pronouncedly serrated (Fig. 2M). The assignment to the species *S. walkeri* was based on the presence of two longitudinal rows of five prominent tubercles flanked on either side by a shorter longitudinal row of three prominent tubercles on the dorsal surface of pleotelson, two on either side of midline (Fig. 1K). This character is also reported from the Persian Gulf specimens (Khalaji-Pirbalouty & Wägele, 2010), Tunisian ones (Ben Amor, Rifi & Ben Soussi, 2015) and Africa ones (Jacobs, 1987). The pleotelson is long and tapers to a rounded point that is slightly upturned; margin of telson crenated. Endopod of uropod has dorsally prominent, median tubercles, and exopod with five to six large, triangular, external teeth plus an acute apex of the exopod (Fig. 1M), as other authors pointed out (Pillai, 1955; Harrison & Holdich, 1982; Ben Amor, Rifi & Ben Soussi, 2015). The number of teeth varies also within the same individual. The number of articles in the antenna flagellum varies, depending on size, and bears a fringe of smooth setae at the distal interior angle, in female reaching only as far as end of next segment (Fig. 2L).

Ecological remarks: This species is a shallow, warm-water, fully marine isopod common in crevices and in fouling. Occasionally, it has been recorded as a wood-boring species (Khalaji-Pirbalouty & Wägele, 2010); however, it is to be noticed that traces of wood have not been found in the stomach contents of this species and its mouthpart morphology is not that of a true wood-boring sphaeromatid (see Carlton & Iverson, 1981). Instead, these authors suggest a thigmotactic response. This means *S. walkeri* has a predilection for holes and crevices, which explains its occasional observations in wood, benthic algae, stones, dead sea squirts, mangrove roots, empty barnacle shells like those of *Balanus amphitrite* Darwin, 1854, oscula of sponges and dead ascidians including *Ciona intestinalis* (Ben Amor, Ben Slaem & Ben Souissi, 2010; Ben Amor, Rifi & Ben Soussi, 2015). It is a thermophilic isopod, with high densities during spring and summer. Its reproductive biology was positively correlated with salinity, transparency of water and temperature, and it breads continuously throughout the year in some introduced populations (see Ben Amor, Rifi & Ben Soussi, 2015).

## Discussion

At present, 12 marine exotic isopod species are known to be present in European waters. Ten of them are free-living species, most of them considered to be established, and two are parasites and considered to be casual (Streftaris, Zenetos & Papathanassiou, 2005; Zenetos et al., 2010; Galil, 2011; Noël, 2011; Lavesque et al., 2013; Chainho et al., 2015; Lorenti et al., 2016; Marchini, Ferrario & Occhipinti-Ambrogi, 2016a; Ulman et al., 2017) (see Table S1). The Iberian Peninsula alone hosts 50% of these ten free-living species, proving to be an important monitoring point for spread as well as future arrivals of exotics. Moreover, 50% of the marinas sampled in 2017 had increased their number of exotic isopods within the timeframe of only six years (Table 1). The case of the marinas in Cádiz Bay (Strait of Gibraltar) is to be noticed. Only *Paracerceis sculpta* was found in 2011, but they hosted *P. sculpta*, *Paradella dianae*, *Sphaeroma walkeri* and *Paranthura japonica* in 2017 (see the case of St. 12, 13 and 14.1 in Table 1). It is to be noticed that, despite more habitat-forming species were analyzed in 2017 in comparison with 2011, the increase in NIS was verified for the same species. In fact, a previous study conducted by Ros et al. (2013) demonstrates that about 50% of the dominant sessile species present throughout the year in Puerto América marina (St. 14.1) are introduced. Several factors may be favouring the introduction and establishment of exotic species in this area. Some of these factors may be due to particular environmental conditions of each marina; but others are most likely human-related, like the proximity of these marinas to a major international port in southern Spain (Cádiz Port), together with the high maritime traffic occurring across the Strait of Gibraltar.

History of introduction, pathways, vectors and potential spread of each species are discussed below.

Histories of introduction and worldwide distribution

*Ianiropsis serricaudis* is native to the western Pacific, from the Sea of Okhotsk to the Sea of Japan, including Russia, Japan and Korea (Kussakin, 1962; Jang & Kwon, 1990; Shimomura, Kato & Kajihara, 2001; Yokoyama & Ishihi, 2007) (Fig. 3A). It was reported as NIS in San Francisco Bay, California (Carlton, 1979) in association with the introduced ascidians *Ciona intestinalis* Linnaeus, 1767 and *Styela clava* Herdman, 1881, possibly transported in shipping associated with the Vietnam War (Carlton, 1979). In the following years, reports of unknown *Ianiropsis* or erroneously identified specimens started to appear in the East and West coast of the United States and in 2004 it was already present in Europe, associated with the introduced ascidian *Syela clava* in Southampton (England) (see Hobbs et al., 2015 and references herein). In the Netherlands it was first observed in 2000 (Faasse, 2007) in an estuary used for shellfish aquaculture, and near the port of Rotterdam, among other locations. In 2010 and 2011, Hobbs and collaborators realized that all the mentioned reports involved the same species, potentially globally distributed by ships. From 2010 to 2013 it was widely reported from Maine to New Jersey (United States, western Atlantic), in association with both native and introduced algae, bryozoans and ascidians from fouling communities on floating dock sites and pilings (Pederson et al., 2005; McIntyre et al., 2013; Janiak & Whitlatch, 2012; Johnson, Winston & Woolacott, 2012; Wells et al., 2014; Hobbs et al., 2015). Also in California and Washington (United States, eastern Pacific), in mudflats near reefs of the introduced Australian serpulid polychaete *Ficopomatus enigmaticus* (Fauvel, 1923) (Heiman & Micheli, 2010) or in association to the non-native tunicate *D. vexillum* colonizing mussel aquaculture facilities (Cordell, Levy & Toft, 2013).

The first evidence of its occurrence in the Mediterranean Sea took place in 2012, when it was found to be abundant in the Lagoon of Venice (Adriatic Sea, Italy) (Marchini, Ferrario & Occhipinti-Ambrogi, 2016a; Marchini, Ferrario & Occhipinti-Ambrogi, 2016b). The Lagoon of Venice is a big center for recreational and commercial harbour as well as flourishing mariculture that hosts a high number of introduced species (Occhipinti-Ambrogi, 2000; Marchini et al., 2015). A couple of years later it was present in Olbia (Ferrario et al., 2017), again a major site for mussel farming which, in fact, imports stocks from Adriatic lagoons (Marchini, Ferrario & Occhipinti-Ambrogi, 2016b); and subsequently in French marinas (Ulman et al., 2017). Our results fill a gap in its distribution, providing the first record for the Iberian Peninsula and the Lusitanian province. We now have evidence that it was already present in 2011 in the North of Spain, in La Graña marina (Ferrol, Galicia). Ferrol city has been a major naval shipbuilding centre for most of its history, and today, aquaculture and fishing stand as its primary industries.

Interestingly, the specimens found in Ferrol bear four marginal denticles on pleotelson (Fig. 1D). There are some minor discrepancies regarding this character; Gurjanova (1936) described it as possessing four or five, Kussakin (1962) established a range of four to seven, Jang & Kwon (1990) showed four on the material from Korea, Doti & Wilson (2010) established “five denticles or more” but not “up to four denticles” and Marchini, Ferrario & Occhipinti-Ambrogi (2016a); Marchini, Ferrario & Occhipinti-Ambrogi (2016b) reported three or four from the specimens collected from the Mediterranean Sea. In any case, Hobbs et al. (2015) considered this to be a variable character and they relied on additional characteristics instead. They suggested a founder effect from the narrower range of denticle counts in introduced populations (three to four) *versus* the reported from native regions (up to seven). Moreover, our specimens were considerably large (males up to 5 mm and ovigerous females up to 3 mm) in comparison to those reported from Russia (2.9 mm for males and 2.7 for females) (Kussakin, 1962, Kussakin, 1988) from the East coast of the United States (largest male being 3.2 mm and female 2.4 mm) (Hobbs et al., 2015) and from the Mediterranean Sea (around 3 mm) (Marchini, Ferrario & Occhipinti-Ambrogi, 2016a; Marchini, Ferrario & Occhipinti-Ambrogi, 2016b). Whether these morphological changes imply changes in the ecological performance of the species in the new range and whether these are the result of changes at the genetic or only phenotypic level are uncertain. The biological, social and economic impact *I. serricaudis* may have in the introduced areas cannot be estimated until further ecological studies are carried out, since there is a severe lack of information for this species, even in its native range (Hobbs et al., 2015).

In the Iberian Peninsula, the arrival of *I. serricaudis* is probably linked to accidental introduction with shellfish transfers. This is a likely associated vector (see Marchini, Ferrario & Occhipinti-Ambrogi, 2016a; Marchini, Ferrario & Occhipinti-Ambrogi, 2016b), judging by the occurrence of the species in European mussel aquaculture facilities and hotspots for mariculture and shellfish trade. In fact, Galicia, together with Cataluña, bear the highest oysters, clams and mussel production of Spain, including production of non-native species such as the Pacific oyster (*Crassostrea gigas*) and the Japanese clam (*Ruditapes philippinarum*), and export to other countries of Europe (Instituto Galego de Estatistica, 2017; Ministerio de Agricultura y pesca, 2017). This vector has been attributed to several species with similar routes of introduction (see Gruet, Héral & Robert, 1976), including the isopod *Paranthura japonica* (see Figs. 3A, 3E) (Lavesque et al., 2013). Nevertheless, shipping transport is an associated vector of this species as well, given its presence in ports and its nature as fouling species of hard substrates such as docks, as well as its adaptability to different substrates (Hobbs et al., 2015). Our finding in a marina adds recreational boating as a vector, at least, for secondary transport. This means *I. serricaudis* has the potential to spread to further Mediterranean marinas as well as along the Iberian Peninsula coast. This would be not surprising since this species bears broad temperature tolerance and is expected to spread through Europe as was well as temperate waters of the southern hemisphere (see Hobbs et al., 2015). As a small-size organism, it is likely to be overlooked though; therefore, we call for prevention for the detection of this species in the mentioned areas.

*Paracerceis sculpta* is the most widespread species within the genus and a successful species colonizing new areas. Its type locality is San Clemente Island, California (USA) (Menzies, 1962) and its putative native range includes the northeastern Pacific region, including California (Richardson, 1905; Wallerstein, 1980; Austin, 1985; Reed & Hovel, 2006), San Quintin Bay, southern Baja California (Menzies, 1962); Puerto Peñasco, Sonora (Ohmart, 1964) and northern and central Gulf of California (Mexico) (Brusca, 1980). It has been present in Hawaii at least since 1943, probably introduced by naval shipping from southern California (Miller, 1968; McCain, 1975); and at least since 1978 in Brazil (Pires, 1980a; Pires, 1981; Loyola e Silva, Masunari & Dubiaski-Silva, 1999). It was only detected from the Gulf of Mexico in 2009 (Munguia & Shuster, 2013). From the 1990s onwards, it was reported from distant locations; from China (Yu & Li, 2001), Hong Kong (Bruce, 1990; Yu & Li, 2003), Taiwan (Yu & Li, 2003) and Japan (Ariyama & Otani, 2004), to Australia (Hass & Knott, 2000; Hewitt & Campbell, 2001) and northwest Indian Ocean, in Pakistan (Yasmeen & Yousuf, 2006). It is also considered introduced in South Africa, being ship fouling and/or ballast water its associated vector (Barnard, 1940; Griffiths, Robinson & Mead, 2009; Mead et al., 2011). In the Mediterranean Sea, it is known from the central region since the 1970s (Rezig, 1978; Forniz & Sconfietti, 1983; Forniz & Maggiore, 1985; Lombardo, 1985; Savini et al., 2006; Cosentino, Giacobbe & Potoschi, 2009; Vincenzi et al., 2013), and decades after it was reported from the eastern (Katsanevakis et al., 2014) and western Mediterranean as well (Marchini, Ferrario & Minchin, 2015). In the eastern Atlantic Ocean it was found for the first time in 1988–1989 in Cádiz bay (Spain) (Rodríguez, Drake & Arias, 1992). In the Macaronesia biogeographical region (northwestern Africa) it was detected only in 2014 (Marchini et al., 2017), collected from Ponta Delgada marina (Azores archipelago) and in 2015 (Ramalhosa et al., 2017), collected from Funchal marina (Madeira archipelago).

There is evidence for attributing shipping, including recreational boating, as vector to *Paracerceis sculpta* (Hewitt et al., 2004; Katsanevakis et al., 2014; Mead et al., 2011; Marchini et al., 2017). It is commonly found in locations of intense vessel traffic; in marinas, bays or coastal lagoons near major harbor facilities (Rezig, 1978; Forniz & Sconfietti, 1983; Rodríguez, Drake & Arias, 1992; Castelló & Carballo, 2001; Espinosa-Pérez & Hendrickx, 2002; Marchini et al., 2017). In the 1990s it was already present in the Mediterranean Sea and the Strait of Gibraltar. From there, it has been subsequently found in additional marinas along the southern and eastern sides of the Iberian Peninsula coast from 2011 to 2017 (Table 1); and it currently occurs from southern Portugal to eastern Spain. We report it for the first time for Alboran sea ecoregion, where all the males found belonged to the alpha morph *sensu* Shuster (1992). This supports the idea that only the alpha morph has made it into the introduced populations, consistent with the lack of beta and gamma male records in other non-native locations (Pires, 1981; Forniz & Maggiore, 1985; Rodríguez, Drake & Arias, 1992; Loyola e Silva, Masunari & Dubiaski-Silva, 1999; Hewitt & Campbell, 2001; Yu & Li, 2001; Ariyama & Otani, 2004; Munguia & Shuster, 2013; Marchini et al., 2017). In fact, Shuster & Wade (1991) hypothesized that the shorter lifespan of beta and gamma males is a handicap for surviving long trips and colonizing new regions.

In the Iberian Peninsula, *Paracerceis sculpta* is mainly associated to the introduced/cryptogenic bryozoan *Bugula neritina* and the introduced *A. verticillata*, which may have facilitated the transport and establishment of this exotic isopod (Marchini, Ferrario & Minchin, 2015; Marchini et al., 2017; Gavira-O Neill, Guerra-García & Moreira, 2016). Additionally, we have observed a non-overlapping presence of *P. sculpta* and the native isopod *Dynamene edwardsii* in most of the stations. A further study investigating the interspecific interaction of these two species is scheduled, in order to determine the potential biological impact of *Paracerceis sculpta*.

Similar to *Paracerceis sculpta*, *Paradella dianae* was first reported from Bahia de San Quintin, Baja California and Mexico (Menzies, 1962). Its native range is supposed to be Northeast Pacific, from Ventura County (California, USA) to Michoacán (Mexico), including the Gulf of California (Iverson, 1974; García-Guerrero & Hendrickx, 2004) (Fig. 3C). Before the 1980s it was reported in the western Atlantic in Puerto Rico (Menzies & Glynn, 1968) and Brazil (Pires, 1980b). First record outside of its native range was in Marshall Islands in 1967 (Glynn, 1970). From the 1980s onwards, it was found in distant areas of the world. In western Pacific, in Hong Kong in 1986 (Bruce, 1990); in Australia (Harrison & Holdich, 1982; Furlani, 1996; Hass & Knott, 2000), collected from small boats jetties; and at the other side of Indian Ocean in Pakistan (Arabian Sea) in 1984 (Javed & Ahmed, 1987). At the same time, *Paradella dianae* arrived to the southeastern coast of USA (western Atlantic) (Clark & Robertson, 1982; Van Dolah, Knott & Calder, 1984; Fox & Ruppert, 1985; Kensley & Schotte, 1989; Nelson & Demetriades, 1992), being ship fouling the most likely vector (Knott & De Victor 2010).

It is unknown whether *P. dianae* arrived to the Iberian Peninsula and the Mediterranean Sea from the Indian Ocean, from the Atlantic Ocean, or from both through multiple introductions. It was reported from the Italian coast in 1980 (Forniz & Maggiore, 1985) and the coast of Alexandria (Egipt) (Atta, 1991); but at the same time reported across the Strait of Gibraltar, in Cádiz Bay (Atlantic side of the Strait) in 1988–1989 (Rodríguez, Drake & Arias, 1992) and Algeciras Bay (Mediterranean side of the Strait) in 1992 (Castelló & Carballo, 2001). From 2000 onwards it was collected and reported from additional locations in Central Mediterranean Sea (Bey et al., 2001; Ferrario et al., 2017; Ulman et al., 2017); and Eastern Mediterranean Sea (Zgozi, Haddoud & Rough, 2002, Kirkim et al., 2010; Çinar et al., 2008, Doğan, Bakir & Katağan, 2015, Kirkim, Özcan & Katagan, 2015; Ulman et al., 2017).

As well as *P. sculpta*, it was probably introduced to new locations by hitchhiking on the hulls or other surfaces of ships (Rodríguez, Drake & Arias, 1992; Galil, 2011). Hass & Knott (2000) also point to recreational boating as a likely vector, at least for its introduction to Australia. Our study supports this hypothesis, since it was found again in marinas located in Cádiz Bay (Strait of Gibraltar’s vicinity) plus others along the Alboran Sea coast. Marinas of southern Iberian Peninsula coasts are well connected by frequent local traffic; 90% of visiting boats in the sampled marinas are Spanish, plus a percentage of foreign boats usually coming from Europe (UK, France, Holland) and other parts of the world (America, Australia, Arabic countries) (marina staff, personal communication). In fact, our data shows an ongoing expansion of *Paradella dianae* into additional marinas, potentially colonizing the eastern side of the Iberian Peninsula into the western Mediterranean Sea. Even having the same native range and potentially bearing a similar pattern of introduction than *P. sculpta*, *P. dianae* does not seem to be as successful, bearing lower densities than *P. sculpta* and a smaller introduction range (Figs. 3B, 3C).

*Sphaeroma walkeri* is the most widespread of these species, reaching numerous ports worldwide (see Carlton & Iverson, 1981). Stebbing (1905) first described it from in Ceylon (now Sri Lanka, Indian Ocean), with the northern Indian Ocean being its native range, including India, Arabian Sea and Bay of Bengal (Carlton & Iverson, 1981). It was known from the Persian Gulf some years later and the introduction status in this locality is doubtful, thus considered cryptogenic (Fofonoff et al., 2017) (Fig. 3D). Carlton & Iverson (1981) propose an episodic dispersal for this species. An initial local transport (pre-1870 period) would have occurred around the Indian Ocean plus South Africa (Stebbing, 1917), where it was found in fouling on pilings, Mozambique (Barnard, 1955) and Australia (Baker, 1928; McNeill, 1932; Iredale, Johnson & McNeill, 1932). A second period would be related to the opening of the Suez Canal in 1869. The record of this species in Port of Suez already in 1904–1905 (Stebbing, 1910) is doubtful; therefore, we agree with Fofonoff et al. (2017) and consider *S. walkeri* cryptogenic from this locality as well (Fig. 3D). From there, it would have travelled through the Suez Canal into the Mediterranean Sea (Omer-Cooper, 1927; Larwood, 1940). A post 1940 period would have been coincident with World War II*. Sphaeroma walkeri* would have been transported to the American continent associated to the intense shipping traffic since that time. It was found in Brazil (Loyola e Silva, 1960), Puerto Rico (Menzies & Glynn, 1968), Florida (Miller, 1968; Camp, Whitino & Martin, 1977; Nelson & Demetriades, 1992) and Hawaii (Miller, 1968). From those areas, it continued to increase its distribution to different parts of the world. To the western Pacific in Hong Kong in 1972 (Vrijmoed, 1975; Morton, 1987), Hainan (southern China) from pier fouling samples (Kussakin & Malyutina, 1993) and other locations in Australia (National Museum of Natural History (Smithsonian Institution) collections (NMNH), 1967; Montelli & Lewis, 2008). To the eastern Pacific in San Diego Bay (California), it was first detected in 1973 in fouling on pilings, floats and small boats at yacht harbours (Carlton & Iverson, 1981). Along the western Atlantic coast it was found in other locations of the Gulf of Mexico (Clark & Robertson, 1982; Cházaro-Olvera et al., 2002), Cuba in 1994 (USNM 280039, US National Museum of Natural History 2007) and Isla Margarita (Venezuela) in 2004 (Gutiérrez, 2012). Along the Northwest coast of Africa, it was also associated with harbours (Jacobs, 1987). On the Indian Ocean it was reported from Malaysia only in the 1990s (Rai-Singh & Sasekumar, 1996) and from Iran in 2006–2010 (Khalaji-Pirbalouty & Wägele, 2010). Across the Mediterranean Sea, it continued spreading to further eastern locations until the present year (Glynn, 1972; Kocataş, 1978; Galil, 2008; Ulman et al., 2017). It was recorded in the Italian Peninsula (Lodola, 2013) and found to be completely established with successful populations in Tunisia harbours and lagoons (Ben Souissi et al., 2004; Ben Amor, Ben Slaem & Ben Souissi, 2010). In was also reported in the western Mediterranean (Zibrowius, 1992), being reported from Spain for the first time in 1981 (Jacobs, 1987). In 2017, we report *Sphaeroma walkeri* from the southern Iberian Peninsula, in Cádiz Bay.

The route of introduction to southern Spain and the Strait of Gibraltar is unknown and several are possible. Initially, specimens may have arrived to the Mediterranean Sea from faraway ports in Indian Ocean or Australia; or from the long-established population in Suez Canal, and subsequently spread towards the western Mediterranean Sea, arriving to France and eastern Spain. It may also have arrived from western Atlantic populations from America or northwestern Africa and entered through the Strait of Gibraltar (Spanier & Galil, 1991; Galil, 2008); or from both Indian and Atlantic populations through multiple introduction events. In any case, its presence in Puerto América marina also indicates a transport via shipping, including recreational boating as vector. This supports the findings of Ulman et al. (2017), who collected individuals of *S. walkeri* directly from hull fouling of recreational vessels in Mediterranean marinas. Interestingly, *S. walkeri* was first reported from the Macaronesia biogeographical region only two years ago; at Funchal marina, presumably introduced by means of recreational boating from populations in the Canary Islands (Spain) or the Madeira island system itself (see Ramalhosa et al., 2017). Considering that *S. walkeri* was already present in Marocco and Mauritania (northwestern Africa) since the early 1980s (Jacobs, 1987), it could have introduced to marinas across Madeira, Canary Islands and the Strait of Gibraltar years ago, even though it was detected only now. An interspecific competition pressure among *S. walkeri* and its congener *S. serratum* has been suggested for the Lagoon of Tunis (Ben Amor, Rifi & Ben Soussi, 2015), but further studies are necessary to evaluate its biological impact in the Iberian Peninsula.

Finally, Richardson (1909) first described *Paranthura japonica* from material collected from Muroran (North Japan). Its native range only includes localities from Japanaese coasts (Nunomura, 1977; Yamada et al., 2007), eastern Russia (Sea of Japan) (Nunomura, 1975; Moshchenko & Zvyagintsev, 2004), Kurile Islands (Kussakin, 1975) and eastern China (Che & Morton, 1991; Li, 2003; Zhang et al., 2009; Wang, Ren & Xu, 2010) (Fig. 3E). It was reported as alien for San Francisco Bay in 1993 (Cohen & Carlton, 1995) and found to be widespread in southern California harbours in 2000 (Cohen et al., 2005). Between 2007 and 2010 it was first found in European waters; in Arcachon Bay (Bay of Biscay, France), probably introduced with oyster transfers. This Bay is one of the major French oyster farming sites (Verlaque et al., 2008), and during the 1970s, the exotic Pacific cupped oyster *Crassostrea gigas* (Thunberg 1793) from the Senday Bay (Japan) was massively introduced (Mineur et al., 2014), in order to sustain the local industry after a viral disease of *Crassostrea angulata* (Lamarck 1819). *Paranthura japonica* probably remained unnoticed or misidentified since then (see Lavesque et al., 2013). It was found in the Mediterranean for the first time in the Lagoon of Venice, probably in 2000 (Marchini et al., 2014). It is thought to have arrived as shellfish import directly from Arcachon Bay, associated with the clam *Ruditapes philippinarum* (Adams and Reeve 1850) during the 1970s; and secondary spread to further Mediterranean marinas (see Marchini et al., 2014; Marchini, Ferrario & Minchin, 2015; Lorenti et al., 2016; Ferrario et al., 2016b; Dailianis et al., 2016; Tempesti et al., 2016; Ferrario et al., 2017; Ulman et al., 2017).

It was reported only recently from the Iberian Peninsula, from samples collected from fouling assemblages in marinas of the eastern coast in 2016 (Ulman et al., 2017). Nevertheless, our study proves that *P. japonica* has been present in Barcelona and Valencia (eastern Iberian Peninsula) at least since 2011. Ulman et al. (2017) suggest this species to be ‘polyvectic’ (meaning it has been transported by multiple mechanisms, according to Cohen (1977), Carlton & Ruiz (2005)), and points at recreational boating as vector for its secondary spread across the Mediterranean Sea. Our data supports this hypothesis, since *P. japonica* was found in Barcelona, Benicarló and Mallorca (Balearic Islands), which are popular destinations for vessels cruising the western Mediterranean in between Barcelona to the West and northwestern Italy to the East (Ulman, personal communication). In 2014, two individuals of *P. japonica* were found within the Strait of Gibraltar’s vicinity, in Chipiona rocky shores (Cádiz) (Cabezas, pers.comm); and three years later, it was abundant in marinas located in Cádiz Bay. Cádiz is a great hotspot for both international commercial shipping and pleasure craft, as well as a center for aquaculture production, including the Japanese clam *Ruditapes philippinarum* (Junta de Andalucía, 2014). Just as in Italy, this clam was intentionally introduced for commercial use in Spain in the 1970s. Despite having conducted several samplings in Cádiz marinas before 2014, this species was never found to be present before that date. On one hand, it is possible that *P. japonica* has arrived to Cádiz bay due to shellfish transfers since the 1970s, but have remained unnoticed and located only in aquaculture facilities instead of spreading to nearby marinas, thus undetected during sampling campaigns. On the other hand, it seems more likely that it spread via recreational boating from the Italian Peninsula to the eastern Iberian Peninsula (present in 2011), and later on to Cádiz marinas (present in 2017). It is to be noticed that *P. japonica* was not present in the bryozoan *B. neritina* in Puerto América marina in 2011; but it was found associated to the same host in 2017. This fact supports this record as a new arrival of NIS into a particular region, and thus represents a Marine Strategy Framework Directive indicator to establish Cádiz Bay as a hotspot for marine introductions, following Olenin et al. (2016).

## Conclusions

We have reported a distribution range extension for all exotic isopod species present in the studied areas, some of them proving to be polyvectic and well established in marinas. The next step is to evaluate their potential biological, social and economical impact, however, there are gaps of knowledge that hamper this task. Baseline studies delving into the ecology of all these species (*i.e.* role as prey-predator in the trophic chain, habitat selection, role in their ecosystem functioning) are of great need in here (see Table 1 Blackburn et al., 2014). Although none of the NIS found in the present study were found in the extensive survey of natural coastal habitats by Guerra-García et al. (2012), future surveys including natural areas would be necessary to detect a potential secondary spread into these habitats.

There is a critical problem that keeps recurring and needs to be reduced: the lags in detection of a new arrival. In many occasions, much time lapse between the initial introduction and the report of it, with a bias for noticing invaders only after they become an abundant nuisance, due to inadequate monitoring or lack of taxonomic expertise (see Crooks, 2005). This happens often in the case of small-sized and scarcely studied organisms, which often remain overlooked until they reach high densities and the spreading process is advanced. But small does not mean “unimportant” (Carlton, 2011) and, since biological invasion processes are “irritatingly idiosyncratic” (Richardson et al., 2000), exotics can exist in relatively low numbers before exploding. This means we risk underestimating the potential impact of taxa like the Order Isopoda that, as shown in the present study, can subsequently spread across additional marinas within a short timeframe.

In order to be ready for decision making and implementation of invasion control, as well as assessment of future arrivals, prevention is the key; and all this starts with building comprehensive data on the presence and distribution range of exotic species, especially on new arrivals (see Bishop & Hutchings, 2011; Groom et al., 2015; Olenin et al., 2016). We consider this account serves as documentation and update about the marine exotic isopods dwelling in the Iberian Peninsula, a hotspot for exotics arrival; as well as drawing attention to these overlooked organisms and the risk of recreational boating as vector for introduction and secondary spread.

##  Supplemental Information

10.7717/peerj.4408/supp-1 Table S1 Introduced isopod species currently known from European waters List of introduced isopod species in European waters, updated with the findings of the present study. Name of the species, parasite/free-living status, origin, distribution in European waters, introduction status remarks and likely vectors of introduction are provided. MED, Mediterranean Sea; WMED, Western Mediterranean; CMED, Central Mediterranean; EMED, Eastern Mediterranean; ATL, Atlantic; NOR, North Sea; C, casual; E, established; NE, non-established; nd, no data available. Species with asterisk are those found to be present in the Iberian Peninsula.

## Additional Information and Declarations

### Data Availability

The following information was supplied regarding data availability: The specimens described in the manuscript are stored in the Laboratorio de Biología Marina, Department of Zoology, University of Seville (Spain) In addition, the following specimens of each species as voucher material are deposited in Museo Nacional de Ciencias Naturales (MNCN, CSIC), Madrid, Spain: (http://www.mncn.csic.es/): MNCN 20.04/11439: *Ianiropsis serricaudis*. Two males collected from the bryozoan *Bugula neritina*, floating pontoons, in La Graña marina (Ferrol, Spain), 07/05/2011 MNCN 20.04/11440: *Paracerceis sculpta*. Two males and two females collected from fouling substrates, floating structures (pontoons, ropes and buoys) from Faro marina (Faro, Portugal), 26/06/2017 MNCN 20.04/11441: *Paradella dianae*. One male and two females collected from Corallinaceae algae and green algae, floating pontoons, from Rota marina (Cádiz, Spain), 13/05/2017 MNCN 20.04/11442: *Sphaeroma walkeri*. One female collected from fouling community, floating structures (pontoons, ropes, buoys), from Puerto America marina (Cádiz, Spain), 02/07/2017 MNCN 20.04/11443: *Paranthura japonica*. One male and two females collected from fouling community on floating structures, from Puerto America marina (Cádiz, Spain), 02/07/2017.
