# Supplementary material for: Marine exotic isopods from the Iberian Peninsula and nearby waters
Source: PeerJ. 2018 Feb 27;6:e4408. doi: 10.7717/peerj.4408 (PMC5833481; doi:10.7717/peerj.4408)
Supplement: Table S1 — List of introduced isopod species in European waters, updated with the findings of the present study. Name of the species, parasite/free-living status, origin, distribution in European waters, introduction status remarks and likely vectors of introduction are provided. MED, Mediterranean Sea; WMED, Western Mediterranean; CMED, Central Mediterranean; EMED, Eastern Mediterranean; ATL, Atlantic; NOR, North Sea; C, casual; E, established; NE, non-established; nd, no data available. Species with asterisk are those found to be present in the Iberian Peninsula. [file peerj-06-4408-s001.docx]

| Species | Origin | Distribution  (European waters) | Introduction status remarks | Likely vectors of introduction |
| --- | --- | --- | --- | --- |
| Parasites |  |  |  |  |
| *Anilocra pilchardi* Bariche & Trilles, 2006 | Indo-Pacific | WMED, CMED, EMED | C (Zenetos et al., 2010) | Canals (Galil, 2011) |
| *Cymothoa indica* Schioedte et Meinert, 1884 | Indo-Pacific | EMED | C (Zenetos et al., 2010) | Canals (Galil, 2011) |
| Free - living |  |  |  |  |
| *Apanthura sandalensis* Stebbing, 1900 | South Africa | CMED, EMED | E (Zenetos et al., 2010) | nd |
| *Cymodoce fuscina* Shotte & Kensley, 2005 | Persian Gulf | EMED | nd | Vessel fouling (Ulman et al. 2017) |
| ** Ianiropsis serricaudis* Gurjanova, 1936 | NW Pacific | CMED, WMED, ATL (Spain) | E in CMED (Marchini et al. 2016a) and Spain (present study) | Vessels (Ulman et al. 2017; present study) |
| *Limnoria quadripunctata* Holthuis, 1949 | S Indo-Pacific | ATL (British Isles to Spain) | NE in Portugal (Chainho et al. 2015) | Vessel fouling (Noël, 2011; Chaino et al. 2015) |
| *Limnoria tripunctata* Menzies, 1951 | S Indo-Pacific | ATL (British Isles), NOR | nd | Vessel fouling (Noël, 2011) |
| ** Paradella dianae* (Menzies, 1962) | NE Pacific | WMED,CMED,EMED, ATL (Spain) | E in CMED,EMED (Zenetos et al. 2010) | Vessel (Galil, 2011); vessel fouling (Ulman et al. 2017; present study) |
| ** Paracerceis sculpta* (Holmes, 1904) | NE Pacific | WMED, CMED,EMED, ATL (Spain) | E in MED (Zenetos et al. 2010) and Spain (present study) | Vessels (Galil, 2011); vessel fouling (Ulman et al. 2017; present study) |
| ** Paranthura japonica* Richardson 1909 | NW Pacific | WMED,CMED,EMED, ATL (France, Spain) | E in CMED (Ulman et al. 2017) | Vessel fouling and shellfish trade (Lavesque et al. 2013; Marchini et al. 2014; Lorenti et al.2016; Ulman et al. 2017; present study) |
| ** Sphaeroma walkeri* Stebbing, 1905 | Indian Ocean | WMED,CMED,EMED, ATL (Spain) | E (Zenetos et al. 2010) | Vessels (Galil, 2011); vessel fouling (Ulman et al. 2017; present study) |
| *Synidotea laticauda* Benedict, 1897 | Japan | ATL (France) | nd | Vessel fouling and mariculture (Noël, 2011) |
